# Implementation and Outcomes of Peer Support Workers in Services for People With Personality Disorder: A Systematic Review and Narrative Synthesis

**DOI:** 10.1002/pmh.70066

**Published:** 2026-03-08

**Authors:** Ruksana Begum‐Meades, Serena Guillemard, Rimple Limbachiya, Eliseus Lean, Fiona Thompson, Mike J. Crawford

**Affiliations:** ^1^ Imperial College London, Division of Psychiatry London UK; ^2^ Department of Psychiatry University of Oxford Oxford UK; ^3^ Central and North West London NHS Foundation Trust London UK; ^4^ West London NHS Trust London UK

**Keywords:** complex emotional needs, lived experience practitioner, peer support worker, personality disorder, stigma

## Abstract

Peer Support Workers have increasingly been deployed within mental health services to provide support and empower those that may be experiencing challenges of a similar nature to them. While the evidence base is growing, less is known about peer support for people with personality disorder. A systematic review was carried out to identify any existing literature on Peer Support Workers in services for people with personality disorder (Prospero ID: 589614). Five databases were searched (MEDLINE, Embase, PsycINFO, PubMed and CINAHL) from inception to 15 August 2025. Data extracted from relevant studies included country of study, participant sample, study aim and design, any outcome measures used and key findings, which were then evaluated together using a narrative synthesis approach. Thirteen papers were identified. Included studies suggested that Peer Support Workers took on varied roles, including facilitating support groups and advocating for service users. Barriers and facilitators to implementation included being provided with sufficient supervision, as well as receiving training and the ability to maintain boundaries. Studies also assessed outcomes of deployment on service users, including improvement in personality disorder‐related symptoms, increased satisfaction with services and feeling validated and empowered. Initial findings suggest that the implementation of Peer Support Workers in services for people with personality disorder could be valuable if it is done intentionally and meaningfully. Further research is required to strengthen the evidence base in this area, with larger and more diverse samples to work towards enhancing and improving care for people with personality disorder.

## 1 Introduction

Peer Support Workers (PSW) are people who use their lived experience of mental health and recovery to support others. PSWs are increasingly being employed within health services to provide support and empowerment to others who may be experiencing similar challenges to them (Puschner et al. 2019). It has been suggested that through mutuality and reciprocity, PSWs have a unique ability to draw on their own personal experiences when providing support, which allows them to relate to and better understand and empathise with the other individual (Mead and MacNeil 2006). PSWs may be able to help improve continuity of care and enhance access to mental health support for people with mental health difficulties at a time when there is a shortage of professionally trained staff (Gillard et al. 2022; Mallorie 2024).

A growing body of research has examined both the process and outcomes of interventions delivered by PSWs in mental health services (Repper and Watson 2012; White et al. 2020). Qualitative studies suggest that the organisational structure of services in which PSWs are deployed plays an important part in the successful integration and implementation of peer support within a service (Ibrahim et al. 2020). Furthermore, data from randomised controlled trial (RCT) interventions delivered by PSWs show evidence of improved self‐reported recovery, as well as increased empowerment and reduced use of inpatient services (Johnson et al. 2018; White et al. 2020).

The value of peer support for people with a diagnosis of personality disorder has also been considered (Ng et al. 2020). Personality disorder is characterised by enduring patterns of inner experience and behaviour that differ significantly from cultural norms (American Psychiatric Association 2013). People with personality disorder often struggle with forming and maintaining relationships and have negative self‐views, which reduces their quality of life. While there are a number of effective psychological treatment programmes for people with personality disorder (Cristea et al. 2017), access to these programmes is limited, and many are unable or unwilling to engage in them (Crawford et al. 2007; McMurran et al. 2010). As in other areas of mental health, peer support has the potential to increase patient choice and widen access to services, with service users and providers also emphasising the need for greater availability of PSWs for people with personality disorder (Ng et al. 2020).

While there is a growing body of evidence about how, why and where PSWs can best be deployed within general mental health services, less is known about factors that support and impede the delivery of effective peer support for people with a diagnosis of personality disorder. The outcomes of such support are also unclear. We therefore conducted a systematic review to examine the barriers and facilitators associated with the provision of peer support to people with personality disorder, and the outcomes associated with this.

The objectives of the systematic review were as follows:

1. To identify ways in which PSWs have been used to support people with personality disorder.
2. To identify factors that support and impede the deployment of PSWs in services for people with personality disorder.
3. To examine the impact of PSWs on the mental health, service utilisation and well‐being of people with personality disorder.

## 2 Methods

The systematic review was preregistered on Prospero (Prospero ID: 589614).

We search five bibliographic databases (MEDLINE, Embase, PsycINFO, PubMed and CINAHL) from inception to 15 August 2025. Search terms used were (‘peer$’ OR ‘expert by experience$’ OR ‘expert patient$’) AND (‘personality disorder$’ OR ‘complex emotional needs$’) (see Appendix S1). The reference lists of included studies were also manually searched to identify any further eligible papers.

### 2.1 Eligibility Criteria

The SPIDER framework was used to define the key elements of the review question (Cooke et al. 2012):

Sample: Eligible studies needed to either include patients with a personality disorder, even if as part of a larger sample, clinicians who have worked with PSWs with patients with personality disorder or PSWs with experience of personality difficulty who are now supporting people with a diagnosis of personality disorder. PSWs are individuals who have lived experience of mental health challenges and recovery, who are now using this experience to support others experiencing similar difficulties. Within the context of this review, PSWs will have lived experience of personality disorder.

Phenomenon of interest: The process of deploying, ways in which they have supported and/or the impact of PSWs providing support to people with a personality disorder.

Design: We did not restrict studies based on their design, provided they had a clear, stated aim.

Evaluation: Barriers and facilitators, experiences and impact/outcomes.

Research type: Qualitative, quantitative or mixed‐methods studies could be included and must have been published in English.

### 2.2 Data Extraction and Synthesis

One reviewer examined the title and abstracts of the records identified following de‐duplication (RBM). Following this, two reviewers (RBM and SG) independently examined the full‐text papers identified following the search of bibliographic databases and title and abstract screening by RBM. Any conflicts occurring in decisions to include were then reconciled by RBM and SG once full‐text screening was completed. Both reviewers also independently extracted data from included studies, as well as completing quality assessment for these studies. Data extraction and quality assessment were completed on Covidence (www.covidence.org) by both reviewers. Again, any conflicts were reconciled by RBM and SG in a meeting upon completion of these stages. A third reviewer was also available for any conflicts, which could not be reconciled (MC); however, this was not needed.

Data were extracted by two reviewers verbatim from the included studies. Information extracted included title and author(s), country of study, aim of the study, study design, measures used, sample information including inclusion and exclusion criteria, key findings and study limitations. Due to heterogeneity among the included studies, a narrative synthesis approach was used. This allowed us to identify relevant themes across the diverse range of study types and to provide a comprehensive overview of current research evidence on peer support interventions for people with personality disorder, whilst also identifying where there is a need for further research (Popay et al. 2006).

### 2.3 Assessment of Bias

The quality of the included studies was assessed depending on the study design, using one of the Joanna Briggs Institute critical appraisal checklists for qualitative studies, cross‐sectional studies or RCT (Barker et al. 2023; Lockwood et al. 2015; Moola et al. 2020).

## 3 Results

**FIGURE 1:**
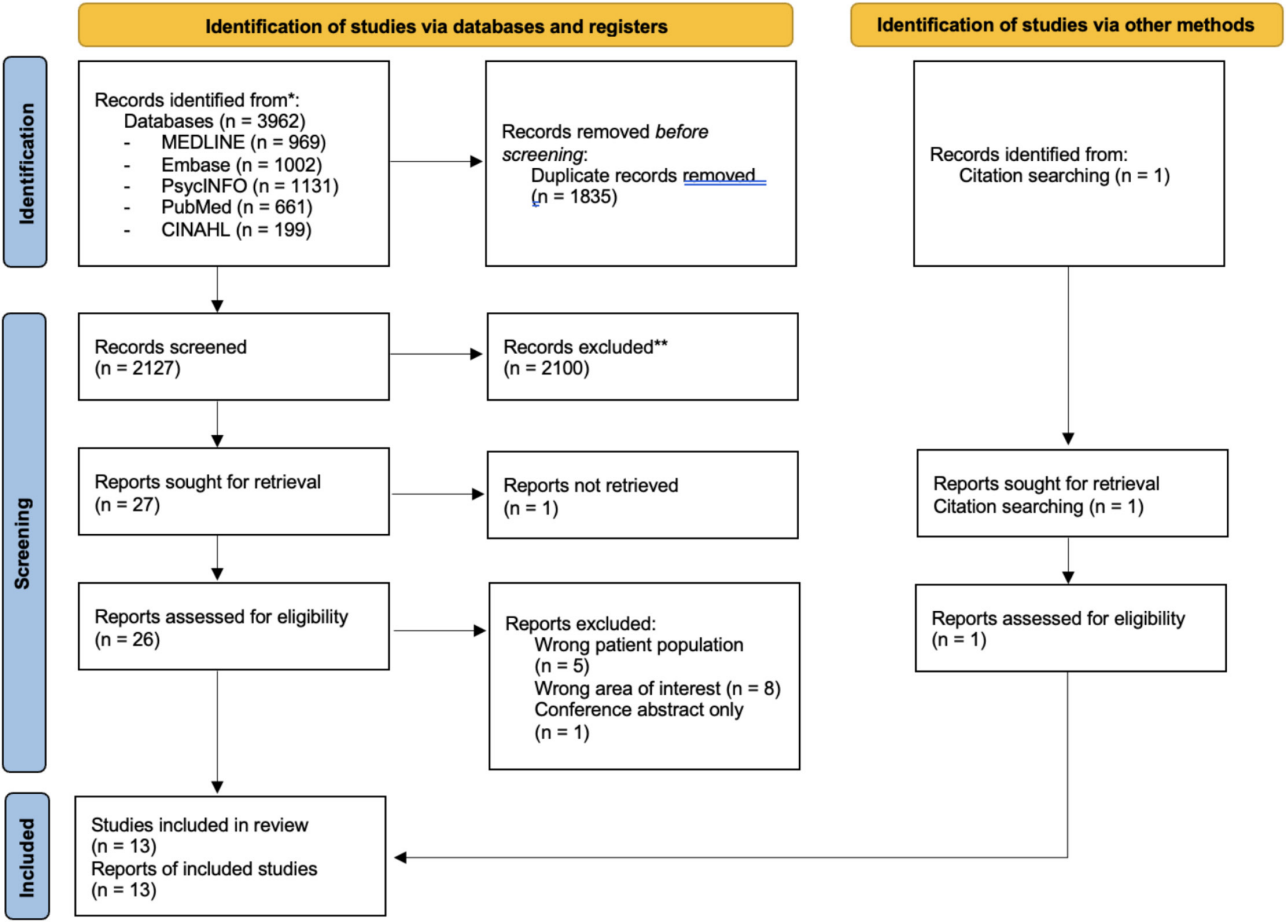
PRISMA flow diagram.

The search of bibliographic databases identified 3962 papers that were potentially eligible for inclusion. Once duplicates were removed, 1835 papers were screened by title and abstract. Of these studies, 27 papers were eligible for full‐text screening, one of which was not available for retrieval. The full texts of the available 26 papers were examined, of which 12 were eligible for inclusion. One additional paper was identified as eligible by manually searching reference lists of included papers, resulting in 13 studies for inclusion in the review (see Figure 1).

### 3.1 Study Characteristics Overview

The studies included in the review were published between 2007 and 2025 (see Table 1). Six studies were conducted in Australia, three in the United Kingdom and one in the United States, Canada, France and Germany, respectively. Seven papers reported the results of qualitative studies, two were uncontrolled pre–post studies, two were mixed‐methods studies incorporating surveys and two studies reported the results of a RCT. Participants consisted of service users with a diagnosis of personality disorder, PSWs with experience of personality difficulty (i.e., those who have lived experienced of being diagnosed with, and treated for, personality disorder) and clinicians working in teams that employed PSWs.

**TABLE 1:** Characteristics of included studies.

Title/authors	Country	Study aims	Sampling	Measures used	Key findings	Study limitations
Randomised controlled trials						
A randomised controlled trial of a peer and clinician led group programme for borderline personality disorder (Grenyer et al. 2025)	Australia	To explore the effectiveness of a manualised brief (6 weeks) co‐facilitated peer and clinician group therapy programme for consumers with BPD.	*Consumers (n = 83); intervention group (n = 43), TAU group (n = 40); female (n = 66); male (n = 7); non‐binary (n = 2); missing data (n = 8)* Recruited through adverts posted in PD interest groups or through direct referral by their healthcare provider.	BPD symptom severity using DSM‐5 criteria Mental Health Inventory‐5 (Berwick et al. 1991)	Participants in the peer co‐led programme showed improvement in BPD symptom severity and mental health. There was a significant decrease in the number of participants that met the DSM‐5 criteria for BPD. 93% satisfaction with treatment in trial arm. 97% of participants rated that they would recommend the treatment to others.	Treatment as usual was used at the comparison arm in this RCT, meaning that the intervention could not be compared to an existing evidence‐based intervention. Majority female sample. There was a 30% dropout rate of participants in the intervention group. All of the peer groups were run online instead of face‐to‐face due to the coronavirus pandemic.
Effectiveness of one‐to‐one peer support for patients with severe mental illness—A randomised controlled trial (Mahlke et al. 2017)	Germany	To compare one‐to‐one peer support with treatment as usual.	*Consumers (n = 216, 23% with PD)* Recruited from services from four hospitals in Hamburg, Germany. Eligible patients had a primary diagnosis of schizophrenia, affective disorders or personality disorder, with a duration of illness or more than 2 years.	General Self‐Efficacy Scale (Schwarzer et al. 1995) Modular System for Quality of Life (Pukrop et al. 2000) EuroQoL Questionnaire—EQUATION 5D (Herdman et al. 2011) Global Assessment of Functioning Scale (Aas 2010)	Self‐efficacy scores at 6‐month follow‐up significantly better in intervention group compared to treatment as usual. A more positive quality of life was found for the intervention group.	Low rate of clinicians ratings making the results of clinical outcomes difficult to interpret. Systematically collected data on further services accessed are also missing. The study was not sufficiently powered to detect differences in secondary outcomes.
Uncontrolled pre–post studies						
Peer support and group psychoeducation for patients with borderline personality disorder: A feasibility study (Blay et al. 2025)	France	To assess the feasibility and acceptability of incorporating a PSW as a co‐leader in an evidence‐based psychoeducation group for BPD.	*Consumers (n = 46)* Recruited from patients who are undergoing a BPD‐focused psychoeducation group.	Zanarini Rating Scale for Borderline Personality Disorder (ZAN‐BPD; Zanarini 2003) Sheehan Disability Scale (SDS; Sheehan et al. 1996) Questionnaire de Fonctionnement Social (QFS; Zanello et al. 2006) Satisfaction form	Significant decrease in ZAN‐BPD score, which was maintained at 1‐month follow‐up. Significant increase in the level of satisfaction in social functioning at 1‐month follow‐up. 82% agreed presence of the PSW made them feel more understood. 68% agreed that examples given by PSWs were more useful than those given by therapists. 82% agreed PSW made them feel more optimistic about the future. Participants attributed up to 82.35% of their benefit from the group to the presence of a PSW.	Lack of control group in the study. PSW in the study was trained, so the results may not be comparable to untrained PSWs delivering interventions.
Evaluation of a skills‐based peer‐led art therapy online‐group for people with emotional dysregulation (Jewell et al. 2022)	Australia	To evaluate an online arts‐based skills programme for people living with BPD, developed and led by a PSW.	*Consumers (n = 31)* Recruited by contacting personality disorder services, advocacy groups and research centres in Australia.	Difficulties in Emotion Regulation Scale (DERS; Gratz and Roemer 2015)	71.1% agreed the programme being run by PSWs made it more likely for them to participate, 10.5% stated this was the only reason they registered. PSW facilitated validation, authenticity and increased understanding. Participants reported that the lived experience perspectives on how skills could be applied were useful to increasing understanding, as well as validating their experiences. Significant reduction in DERS total score over time (*F*(2.52,34) = 34.05, *p* < 0.001).	Small sample size limited the generalisability of the findings. Psychiatric diagnosis was not formally assessed by study researchers. ~50% of participants engaged in art making prior to the programme, so it is unclear if this previous experience impacted the evaluation and acceptability of the programme.
Cross‐sectional studies						
Moving to the other side of the desk: An examination of the practice of including peer support specialists as treatment providers within the dialectical behaviour therapy paradigm (Cawood 2012)	United States	To examine the roles and experiences of DBT peer providers, as well as the DBT therapists who work alongside them.	*PSWs (n = 19); DBT therapists (n = 37)* Purposively recruited via email following attendance at a DBT peer support specialist training event. Invited participants had to be employed at a community mental health (CMH) service across Michigan	DBT PSS Experience Questionnaire McLean Screening Instruments for BPD (Zanarini et al. 2003) DBT Therapist Experience with PSS Questionnaire Maslach Burnout Inventory (Maslach and Jackson 1981)	94.5% PSWs liked being able to use their experiences to help others, and 89% found it rewarding and of personal benefit. 81% DBT therapists had increased hope for patient recovery and believed PSWs helped patients see recovery is possible. Expressed some concerns around PSWs emotional vulnerability and stability.	Consumer perspectives not examined. Both samples used were limited, although the pool of potential participated was also restricted. Rate of participation itself was high. Unclear whether the sample would be generalisable to non‐CMH DBT clinics.
Peer support for borderline personality disorder: A critical review of its feasibility, acceptability and alignment with concepts of recovery (Turner et al. 2024)	Canada	To examine whether peer support could be a viable avenue for supporting more positive, hopeful healthcare encounters for people living with BPD.	*Providers (n = 7)* Programmes for which peer support was a large component or an exclusive focus of their activities.	N/A	PSWs found to generate hope, model recovery, develop a sense of trust, understanding and validation and increase clinician understanding. Clinicians valued PSWs, reducing clinician burnout and increasing patient engagement. Concerns expressed around PSW burnout, lack of training, working beyond their remit, difficulties establishing boundaries and emotional stability. Challenges were also reported in terms of finding and retaining qualified PSWs, as well as demand for services.	Recruitment for the survey engaged primarily charitable peer support organisations, suggesting that the recruitment methods did not reach or engage services from public health systems that are also implementing peer support programmes.
Qualitative studies						
Peer support for consumers with borderline personality disorder: A qualitative study (Barr et al. 2022)	Australia	To investigate the peer worker role in BPD services from the perspectives of peer workers, consumers and clinicians.	*PSWs (n = 5); clinicians (n = 4); consumers (n = 14)* Purposive sampling of participants. PSWs needed to have lived experience of BPD.	Characteristics of PS service Experiences of peer support	Consumers and PSWs believed PSWs promoted hope and skills, were more relatable than clinicians and led to consumers feeling less isolated. Clinicians and PSWs discussed reducing stigma and attitude changes, and PSWs feeling valued. However, vicarious trauma, challenges around establishing boundaries, and lack of training for PSWs was also reported.	Small sample size may reflect individual views and are not necessarily generalisable. Majority female sample (all female PSWs and consumers, only one male clinician). Only nurses and occupational therapists were represented in the clinician sample.
Using peer workers with lived experience to support the treatment of borderline personality disorder: A qualitative study of consumer, carer and clinician perspectives (Barr et al. 2020)	Australia	To determine possible models and recommendations of peer support for BPD.	*Consumers (n = 12); clinicians (n = 12); carers (n = 12)* Purposive sample of participants via email, snowballing strategy used.	N/A	PSWs provided hope and connection, are understanding and validating and can act as advocates and/or role models. They can also offer support when transitioning from acute/inpatient to community care. Emphasis on PSWs being able to maintain their own well‐being. Clinicians had differing views on how integrated PSWs should be in clinical teams.	The use of purposive samples may have produced a biased sample. The majority of participants were female, with all consumer participants female.
Learning the lessons: A multimethod evaluation of dedicated community‐based services for people with personality disorder (Crawford et al. 2007)	United Kingdom	To evaluate the 11 pilot community services and capture lessons learning during the initial phase of their development.	*PD services (n = 11)* Purposive sampling of clinical leads, and further snowball sampling to generate further suggestions for interviewees.	N/A	Agreement that services should facilitate access to peer support. Services offering a range of therapeutic options, including peer support, were highlighted. Service users discussed the value of peer support and being able to learn from others who have had similar experiences.	The data that were collected in this qualitative phase only represent a snapshot or circumstances at an early stage of the development of these services.
Experiences of a peer group for people diagnosed with personality disorder: A qualitative interview study (Dahlenburg et al. 2024)	Australia	To review a peer group intervention through the qualitative analysis of post‐group interviews with participating consumers.	*Consumers (n = 22)* Members of a peer group programme for people with lived experience of BPD	N/A	Consumers experience increased confidence and felt more empowered, reduced stigmatisation, and feeling understood and validated, and connected to the PSW facilitating the group. Consumers felt they were able to speak without pressure and felt that they were on equal footing compared to a clinician‐led approach.	Participation was optional, and people who dropped out of the peer group interventions were less likely to be interviewed. 87% of participants were female (13% male).
The effectiveness of the service user consultant role in specialist personality disorder services (D'Sa and Rigby 2011)	United Kingdom	To consider the role or service user consultants across 11 Department of Health personality disorder pilot services.	*PD services (n = 5)* Staff representatives and service user consultants from the five services taking part in the Department of Health PD pilot	N/A	Common concern was whether PSWs could maintain well‐being. PSWs involved in training, co‐facilitating workshops, involvement in service meetings, with the majority being involved in practical tasks such as training and service development. PSWs benefit from increased confidence and self‐esteem. Concerns around boundaries, duty of care and experiencing prejudice.	Just over half of the pilot services agreed to take part in the study, and as such may not be generalisable to other services, which employ PSWs.
Open access support groups for people experiencing personality disorders: Do groups members' experiences reflect the theoretical foundations of the SUN project? (Gillard et al. 2015)	United Kingdom	To indicate where the theoretical model usefully informs therapeutic practice in the SUN Project and where aspects of the model might be refined to improve practice.	*Consumers (n = 38)* Consumers experiencing difficulties associated with having a diagnosis of personality disorder. 74% female, 42% with a primary diagnosis of personality disorder	N/A	PSWs added value to the group, participants reported a sense of acceptance and feeling understood, with an opportunity for social interaction and reduced sense of isolation.	Data analysed for this study were drawn from a larger study on self‐care and mental health, and as such, the data may not be entirely relevant to the aim being considered here.
The emotional labour of peer work: Encountering stigma in mental healthcare spaces (Seal et al. 2024)	Australia	To understand the workplace experiences of peer support workers with a diagnosis of BPD in mental healthcare settings in Australia.	*PSWs (n = 9)*; *male (n = 2); female (n = 5); non‐binary (n = 2)* PSWs had all received a BPD diagnosis prior to interview	N/A	PSWs highlight emotional labour, stigma, feeling devalued and discredited due to their diagnosis and difficulties in establishing a workplace identity and fitting in. PSWs highlighted negative practices that were associated with people with a BPD diagnosis, with concern that they would not be taken seriously if their diagnosis was revealed to colleagues.	Due to the small sample recruited for this study, it was not possible to segment the analysis by intersections such as race or gender.

### 3.2 Description of Included Studies

The first of two randomised trials of peer support to involve people with personality disorder was conducted by Mahlke et al. (2017) in Hamburg, Germany. The team randomised 216 participants with severe mental illness, of whom 51 (23%) had a diagnosis of personality disorder, to one‐to‐one peer support plus treatment as usual, versus treatment as usual alone. The primary outcome was self‐efficacy over 12 months, measured using the General Self‐Efficacy Scale (Schwarzer et al. 1995). Quality of life (using the Modular System for Quality of Life (Pukrop et al. 2000) and EuroQoL (Herdman et al. 2011)) and service use were also examined. More recently, Grenyer et al. (2025) conducted a RCT of people with borderline personality disorder, in which they compared the delivery of a co‐led, peer support intervention plus treatment as usual, to treatment as usual alone. The primary outcome was severity of symptoms of borderline personality disorder at 3 months according to DSM‐5 criteria (American Psychiatric Association 2013). General mental health (according to the Mental Health Inventory‐5; Berwick et al. 1991) and satisfaction with treatment were also assessed. Within the context of these two studies, ‘treatment as usual’ is when a participant continues with the usual care that they receive throughout the course of the trial. For people with personality disorder in these studies, treatment as usual varied, including group therapy, individual therapy, general case management or contact with their regular healthcare provider.

Two other studies collected longitudinal data from people using services that included PSW. Blay et al. (2025) conducted an uncontrolled pre–post study, which examined the feasibility and acceptability of a psychoeducation group for people with borderline personality disorder that was co‐led by a PSW with lived experience. The team assessed the severity of symptoms of borderline personality disorder using the ZAN‐BPD (Zanarini 2003) among 46 participants prior to participation in the group, 34 participants at the end of the group and 30 participants 1‐month post‐participation. They also assessed people's experience and satisfaction with the group. Jewell et al. (2022) carried out a pilot study to evaluate the provision of art therapy for people with BPD, which was developed and led by a PSW. They aimed to investigate differences in emotion regulation difficulties using the Difficulties in Emotion Regulation Scale (Gratz and Roemer 2015) and collected qualitative data examining the impact of the service.

Two studies collected cross‐sectional quantitative data: Cawood (2012) and colleagues reported on the role of PSWs in the provision of DBT to service users with personality disorder. Nineteen PSWs and 37 DBT therapists were recruited to complete a mixed‐methods questionnaire, which aimed to examine the role of PSWs within services, experiences of PSWs and the associated challenges and benefits, as well as the opinions of DBT therapists on the integration of PSWs into teams and care provision.

Turner et al. (2024) conducted a literature review and a survey of staff working at seven specialist services for people with borderline personality disorder. They examined experiences and beliefs about the implementation, feasibility and effectiveness of these services.

Barr et al. (2020, 2022) similarly investigated the role of PSWs in services for people with BPD, considering the perspectives of PSWs, clinicians and service users. Barr et al. (2020) recruited 12 service users with BPD, 12 carers and 12 mental health professionals to take part in semi‐structured interviews to discuss their perspectives of using PSWs to support the treatment of people with BPD. In a subsequent study by Barr et al. (2022), the team recruited five PSWs, 14 service users and four clinicians to complete a qualitative questionnaire on the experiences and impact of peer support.

Gillard et al. (2015) conducted a qualitative study evaluating the utility of the model underlying the Service User Network (SUN) project for people with personality disorder. Thirty‐eight people attending the SUN project were recruited to take part in semi‐structured interviews, with 31 of these participants also followed up and interviewed 9 months later.

Seal et al. (2024) interviewed PSWs with a diagnosis of borderline personality disorder to understand their experiences of working as a PSW. Nine PSWs who had a BPD diagnosis were recruited to take part in interviews in which they discussed their experiences. Similarly, D'Sa and Rigby (2011) sought to examine the role of PSWs in community personality disorder services in the United Kingdom. Representatives from five services took part in interviews in which they discussed the role of the PSW, the benefits associated with the role, as well as any related difficulties.

Dahlenburg et al. (2024) qualitatively evaluated the experiences of individuals who took part in a peer group for people diagnosed with BPD. Finally, Crawford et al. (2007) conducted a qualitative study exploring the organisation and delivery of 11 specialist personality disorder treatment services in England.

Taken collectively, the included studies investigated the role of PSW in services for people with personality disorder, as well as the barriers and facilitators associated with their deployment. These studies used a variety of designs and analyses to better understand and examine the role of PSWs in services, as well as their impact on the outcomes of service users with personality disorders.

### 3.3 Objective 1—Ways in Which PSWs Have Supported People With Personality Disorder

Across the studies that were included, four discussed the roles and responsibilities of PSWs within multidisciplinary teams (MDTs). Roles and responsibilities undertaken appeared to be varied, including facilitating support groups (Gillard et al. 2015; D'Sa and Rigby 2011), co‐leading therapeutic interventions with clinical professionals (Blay et al. 2025; Grenyer et al. 2025), offering validation, compassion and empathy, modelling behaviour and demonstrating to service users that recovery is possible (Turner et al. 2024; D'Sa and Rigby 2011), and being involved in the provision of professional training, as well as contributing to MDT meetings and advocating for service users (Cawood 2012; D'Sa and Rigby 2011).

### 3.4 Objective 2—Factors Which Support and Impede the Deployment of PSW for People With Personality Disorder

Five of the included studies discussed factors that may be examined as barriers or facilitators impacting the deployment of PSWs in services and to provide support for people with personality disorder.

Facilitators of PSW deployment included having adequate provision of supervision and mentoring in the role (D'Sa and Rigby 2011), PSWs enjoying the work that they are doing and finding a sense of meaning from it (Cawood 2012), and a feeling of gratification from being able to use one's lived experience, and subsequently feeling a sense of empowerment in the PSW's own recovery (Turner et al. 2024; Cawood 2012).

On the other hand, some of the barriers associated with the successful deployment and integration of PSWs included stigma in the workplace (Seal et al. 2024; Barr et al. 2022; D'Sa and Rigby 2011), difficulties in establishing and maintaining boundaries (Barr et al. 2022; Cawood 2012; D'Sa and Rigby 2011) and the emotional demands of the role itself on the PSWs (Seal et al. 2024; Turner et al. 2024; Barr et al. 2022; Cawood, 2011; D'Sa and Rigby 2011). Turner et al. (2024) and Barr et al. (2022) also highlighted the lack of standardised training for PSWs as an additional challenge, as well as the need for consistent, robust and standardised supervision requirements, with concerns surrounding accessing crisis support if facing mental health difficulties of their own when working as a PSW reported by Crawford et al. (2007).

### 3.5 Objective 3—The Impact of PSW on the Mental Health, Service Utilisation and Well‐Being of People With Personality Disorder

Using multilevel modelling analysis, data from the only randomised trial of peer support solely for people with personality disorder (Grenyer et al. 2025) showed statistically significant improvements in both symptoms of borderline personality disorder (according to DSM‐5 criteria) and mental health (measured by the Mental Health Inventory‐5) among those offered 12 h of group therapy that was co‐delivered by a person with lived experience, compared to those offered treatment as usual. In their randomised trial of one‐to‐one peer support versus treatment as usual for a wider group of people with severe mental illness, Mahlke et al. (2017) found that those in the active arm of the trial had improved self‐efficacy at 6 months according to the General Self‐Efficacy Scale. No differences were found in other outcomes including quality of life, social functioning and service use. Two other studies examined quantitative data among people with personality disorder who received support from peer workers. Blay et al. (2025) reported that among the 46 people who were offered a place in a psychoeducation group that was co‐facilitated by a PSW, there were high levels of satisfaction with the contribution made by the PSW. The team found statistically significant reductions in symptoms of borderline personality disorder (measured by the ZAN‐BPD) post‐treatment and 1 month after the group had finished. No differences were found in emotion regulation, alexithymia or impulsiveness. In their pre–post evaluation of an 18‐week arts‐based skills group that was led by a peer mental health professional, Jewell et al. (2022) found large, statistically significant decreases in emotion dysregulation (measured by the Difficulties in Emotion Regulation Scale) post‐treatment and high levels of satisfaction with the programme. Validation of emotions and empowerment in recovery was a recurring finding in qualitative studies (Dahlenburg et al. 2024; Barr et al. 2022; Barr et al. 2020), with patients with personality disorder feeling more understood and accepted, less judged and isolated and more able to share their problems and learn from others with lived experience (Crawford et al. 2007). Self‐stigma was also found to reduce with the presence of PSWs on mental health teams (Dahlenburg et al. 2024).

In order to strengthen the synthesis of the findings across objectives, a summary matrix was developed, which integrated identified barriers and facilitators to implementation with observed outcomes (see Table 2). In doing so, the synthesis of findings was able to go beyond reporting on objectives in isolation by highlighting how factors such as organisational dynamics, training for the role and sufficiency of support structures may impact upon the experiences of service users, the well‐being of PSWs and overall service delivery.

**TABLE 2:** Summary matrix of implementation factors and associated outcomes.

Theme	Implementation (facilitators/barriers)	Service user‐level outcomes	PSW‐level outcomes	Service‐level outcomes
**Organisational support and leadership**	Professional stigma towards personality disorder, and by association the PSW, identified as a barrier		↓ Role satisfaction ↓ Role retention ↓ Well‐being	↓ Integration of PSWs across teams
**Training and role preparation**	Lack of standardised training identified as a barrier	↓ Satisfaction with service provision	↓ Role confidence ↑ Burnout	↓ Consistency of service delivery
**Supervision and mentoring within the role**	Regular reflective supervision identified as a facilitator	↓ Risk of escalation in symptoms	↑ Well‐being ↑ Skill development	↑ Quality assurance by providing standardised supervision
**Role clarity and boundaries**	Difficulties in establishing and maintaining boundaries identified as a barrier	↑ Boundary breaches ↓ Service user clarity on PSW role	↓ Understanding of professional identity	↓ Compliance with policies ↓ Integration of PSWs across teams
**Co‐production and service user involvement**	Co‐production in facilitating support groups and delivering professional training identified as a facilitator	↑ Relevance of support provision ↑ Service user empowerment and feeling of validation	↑ PSW empowerment	↑ Grounding of service delivery model in lived experience

### 3.6 Quality Assessment

Quality assessment across the included studies demonstrated varying levels of methodological rigour across several research designs (see Appendix S2). The two RCTs demonstrated adherence to the core principles of randomisation, with both Grenyer et al. (2025) and Mahlke et al. (2017) employing true randomisation. Outcomes were measured consistently and reliably in both studies. However, notable limitations included the absence of blinding, as well as the lack of follow‐up post‐intervention (Mahlke et al. 2017).

The two uncontrolled pre–post studies both carried out multiple pre‐ and post‐intervention outcome measurements (Blay et al. 2025; Jewell et al. 2022). However, the absence of control groups limited the ability to make causal inferences regarding the effectiveness of the interventions in question.

Cross‐sectional studies were generally robust, clearly defining inclusion and exclusion criteria and using appropriate statistical analysis for the outcome measures selected (Cawood 2012; Turner et al. 2024). However, neither study explicitly addressed confounding factors, which could be considered a limitation to interpretability.

Finally, the included qualitative studies were largely consistent in terms of methodological rigour (Barr et al. 2022, 2020; Crawford et al. 2007; Dahlenburg et al. 2024; D'Sa and Rigby 2011; Gillard et al. 2015; Seal et al. 2024). While most of these studies did not report on the positionality of the research, they were all considered ethically appropriate, with clear methods and analysis.

Overall, despite the limitations discussed above, the included studies used generally sound methodological practices, contributing valuable insights into the topic of peer support for people with personality disorder.

## 4 Discussion

This review summarises the extent and type of research that has been published on the use of peer support interventions for people with personality disorder to date. This includes two randomised trials, of which one focused solely on those with a diagnosis of personality disorder. Studies have also examined the acceptability of interventions led by people with lived experience and qualitatively explored factors that promote and hinder the successful deployment of PSW in mental health services. In contrast to the 13 studies included in this review, systematic reviews on peer support interventions for other mental health conditions have demonstrated a much greater evidence base. White et al.'s (2020) systematic review of RCT on one‐to‐one peer support for adults using mental health services found 23 studies reporting on 19 trials, with a total of 3329 participants, and Smit et al. (2023) reviewed 30 RCTs, meta‐analysing a total of 4152 participants. In comparison, the present review only found two RCTs related to peer support interventions for people with personality disorder with a total of 134 participants, with the remainder of the studies reporting on qualitative or descriptive findings. The marked disparity in the volume and scale of RCTs between peer support interventions for people with personality disorder and those for other mental health conditions may be attributed to several factors. First, people with personality disorder often present with complex clinical profiles, including high levels of emotional dysregulation, interpersonal and relational difficulties and other comorbidities (Tyrer et al. 2015). This may lead to added complexity, which makes standardised intervention delivery less feasible. Second, stigma around personality disorder may contribute to a reduced research focus and funding (Sheehan et al. 2016; Zimmerman and Gazarian 2014). Furthermore, as peer support interventions for people with personality disorder are still in early stages of development, many studies remain exploratory or qualitative, aiming first to establish acceptability and feasibility before then progressing onto extensive efficacy trials. This contrasts with other mental health conditions where peer support service delivery models are more established and have already accumulated a strong evidence base. Collectively, these factors highlight the need for research to better understand the unique challenges associated with supporting people with personality disorder, and how peer support interventions could help to address these.

Data from the included studies on the integration of PSWs into services and support for people with personality disorder suggest that, as with other mental health conditions, peer support has the potential to empower and validate service users seeking treatment, demonstrating to them that recovery is possible, and providing a space in which they feel safe and comfortable to discuss their difficulties (Joo et al. 2022). The very small number of quantitative studies we found meant that we had to conduct a narrative synthesis of the findings. This approach allowed us to explore the benefits and impact of peer support and peer support interventions for people with personality disorder, as well as to identify areas of importance for clinical practice.

### 4.1 Objective 1—Ways in Which PSWs Have Been Used To Support People With Personality Disorder

PSWs were found to support people with personality disorder in the form of facilitating support groups. A benefit of this is that service users may feel more comfortable discussing and sharing their experiences if they know that the facilitator of the group has had similar lived experience, making them feel less judged and more understood. Individuals are more likely to attend treatment and participate if it is being facilitated by a PSW, and feel more validated and hopeful when sharing their experiences in such settings (Høgh Egmose et al. 2023).

Research on PSWs co‐leading therapeutic interventions with clinically trained staff is a limited but growing area of inquiry, suggesting that combining lived experience with clinical expertise may be a promising therapeutic approach (Blay et al. 2025; Grenyer et al. 2025). Having a PSW co‐deliver an intervention may create a more trusting environment in which rapport and therapeutic alliance can be more easily developed, whilst having a clinically trained professional present can allow for greater consistency in PSW‐led interventions, especially given that research has demonstrated the lack of a standardised training approach for PSWs (Beales and Wilson 2015; Cronise et al. 2016). Further research overall is needed to be able to identify best practices regarding integrating PSWs into teams and delivering interventions, as well as to assess the longer‐term outcomes associated with such interventions.

PSWs may also contribute towards the provision of professional training for mental health professionals. This can provide a useful perspective to professionals, demonstrating to them that recovery is possible for people with personality disorder, which has historically been seen as ‘untreatable’ despite research demonstrating high remission rates (Gunderson et al. 2011). Furthermore, by prioritising co‐production of such professional training, training is more likely to be grounded in lived experience, and accurate to the experiences of those individuals that mental health professionals seek to treat (Higgins et al. 2018; Mutschler et al. 2022).

In some services, PSWs formed part of MDTs and attended MDT meetings in which patient cases were discussed among the professionals involved in their care. In such circumstances, PSWs could advocate for service users and use their experiences to suggest what may be the best approach to take in an individual's care (Thomas et al. 2023). This can have a positive impact on both the service user and the PSW: The service user feels heard and understood, with their needs being prioritised and validated, and the PSW may feel a sense of satisfaction and gratification due to their ability to advocate for a service user in their time of need and support them in achieving a positive outcome (Høgh Egmose et al. 2023; Smit et al. 2023).

### 4.2 Objective 2—Factors Which Support and Impede the Deployment of PSW for People With Personality Disorder

Several facilitators of successful PSW deployment were identified. One important factor associated with the successful deployment of PSWs for people with personality disorder was the PSW being provided with adequate supervision and mentoring. Having the ability to reflect upon discussions with service users and debrief with a supervisor where necessary, as well as having regular mentoring and the ability to develop skills in their role as a PSW, are important considerations when it comes to the successful integration of PSWs. Not only does this give them a space to debrief, but it can also support PSWs in feeling valued and included within mental health teams (Cooper et al. 2024; Phillips 2021). Providing adequate supervision can also help to prevent burnout in PSWs, ensuring they remain supported and able to do their job safely and effectively (Forbes et al. 2022; Mancini 2018).

Furthermore, PSWs being able to use their lived experience to meaningfully provide support to others was noted as contributing towards feelings of gratification and enjoyment of the work that they are doing. It can contribute towards a sense of empowerment in PSWs as they are able to provide support to another individual. This could in turn support the PSW in their own recovery journey, allowing them to reflect upon their experiences and the progress they have made in their treatment (Poremski et al. 2022). It can also help them to better manage their own mental health, as they are able to reflect on coping strategies which they find effective during times of crisis, as well as actively working on their relationships with others (Poremski et al. 2022). This achievement and empowerment can drive an individual in their role as a PSW, enabling them to better provide support in their role, as well as managing their own mental health.

Some of the recurring barriers noted in the included studies were around the emotional labour associated with the peer support role. The emotional demands associated with working in specialist personality disorder services have already been highlighted in research examining the experiences of professionally trained staff (Eren and Şahin 2016; Moore 2012), and as such, it is unsurprising that this also emerged as a challenge for PSW. However, providing support to individuals who may be experiencing similar mental health difficulties to that of their own may put PSWs at a heightened risk of re‐traumatisation, which could have a negative impact on their own mental health. Supporting people with personality disorder can be emotionally challenging, and PSWs must have robust support systems to ensure that they are well supported. Equally important is PSWs having access to continued professional development in the form of ongoing training and supervision from other lived experience practitioners, to reflect on the emotional labour of using yourself in the work, and to connect with others who are doing the same. Personal resilience is also crucial—ensuring that PSWs have successfully engaged in treatment and have reached a place of stability and remission is important to maintaining their mental well‐being. Having adequate systems and adjustments put in place is essential for the safety and well‐being of both the patient and the PSW.

Boundary difficulties can also hamper the successful deployment and integration of PSWs in services. This may be further complicated by personality disorder being associated with difficulties with interpersonal relationships (Tyrer et al. 2015). While the peer support role inherently requires an element of self‐disclosure, professional boundaries must be maintained. Role ambiguity can play a large part in difficulties associated with boundaries. Not having clear and consistent role descriptors in place can make it difficult for PSWs to navigate relationships both with service users and other clinical staff, increasing the risk of the PSW struggling with their own professional identity and being at greater risk of burnout (Gillard et al. 2022; Scanlan et al. 2020). One strategy to mitigate this may be for services to facilitate regular supervision of PSWs, allowing them to discuss any difficulties they are facing in the work. This can be particularly beneficial in the case where the supervisor is another, more senior lived experience practitioner, who has experience of the nature of the role as well as common difficulties that may be encountered.

Additionally, professional stigma has the potential to undermine the efforts of PSWs and impact the overall effectiveness of the role (Vandewalle et al. 2016; Viking et al. 2022), especially given there being greater stigma towards personality disorder compared to affective or mood disorders (Hazell et al. 2022). The label of personality disorder and its ability to persevere even after a person has recovered or reached a point of stability with their mental health further perpetuates this stigma. This stigma may lead to clinical staff not recognising the valuable contributions and insights PSWs can bring to MDTs, and how these added insights could ultimately improve patient care and lead to a better understanding of the challenges experienced by people with personality disorder.

Further related to the stigma associated with personality disorder is that clinical staff may still view PSW as being unwell, or call into question their competence or ability to cope with the challenging interactions associated with the role, potentially pathologising a PSW's emotional responses, even though they are appropriate given the work that they are undertaking. This can lead to an unsupportive environment in which PSWs feel they are having to ‘prove’ their capability to colleagues, and this professional hierarchy could impede their ability to do their role (Simpson et al. 2018), as well as their job satisfaction. This added pressure in an already challenging role may lead to increased stress, burnout and isolation of the PSW. It is therefore important that stigmatising attitudes within the workplace are challenged, and PSWs are well integrated into MDTs, being given the opportunity to demonstrate the skills and valuable insights they bring. This can be mitigated through preparation sessions for teams before a new PSW takes up their role, and through applying a ‘top down’ approach, whereby lived experience practitioners who have significant professional experience in the field are employed first, at a more senior level, before entry level staff are brought into teams.

Finally, the lack of standardised training can be an issue when integrating PSWs into services. While some PSWs may receive comprehensive training prior to working with service users, others are only provided with a brief introduction prior to this (Charles et al. 2021). This can lead to difficulties in establishing clear boundaries, developing core competencies and ultimately contributing to burnout as PSWs attempt to navigate a complex clinical environment with insufficient training and resources (Simpson et al. 2014). Services should seek to provide clear, standardised training and professional development opportunities specifically tailored towards PSWs, the nature of their role and the challenges they may experience. In doing so, services would also demonstrate their commitment to integrating PSWs in their teams and appreciating the value and unique insight that they bring.

### 4.3 Objective 3—The Impact of PSW on the Mental Health, Service Utilisation and Well‐Being of People With Personality Disorder

Quantitative data on the impact of PSWs for people with personality disorder are very limited. The two randomised trials (Mahlke et al. 2017; Grenyer et al. 2025) published to date that involved people with personality disorder demonstrate that it is feasible to conduct clinical trials of this type of complex intervention. However, these trials included relatively small samples of people with personality disorder and may not have been sufficiently well powered to capture the full benefits of the interventions they tested. Neither study examined whether any of the benefits associated with the active intervention was sustained in the longer term, or collected data on costs and cost‐effectiveness of these interventions. In contrast, we found detailed evidence of the potential benefits of PSW in the qualitative studies that have been conducted. The potential impact of PSWs has been qualitatively explored from the perspectives of service managers, service users and PSWs themselves. PSWs could provide significant benefits to services, reducing stigma and increasing understanding of personality disorder among clinical staff and demonstrating that recovery is possible. Being present in MDT settings can help to challenge preconceived notions and stereotypes held by clinicians. PSWs are also in a position to advocate for service users, ensuring that clinicians take a person‐centred, holistic approach to each case. This can lead to clinicians providing more compassionate care, in which they themselves are hopeful about the service user's ability to recover, reducing the stigma that service users with personality disorder may experience when navigating mental healthcare. Providing recovery‐oriented care can support more positive treatment outcomes for service users (Cawood 2012; Repper and Watson 2012).

Benefits to the service user can come from receiving more informed and compassionate care. As well as this, service users may feel more validated and empowered in their own recovery, being able to view this as being role‐modelled by PSWs. Service users are also more likely to engage in treatment and experience reduced self‐stigma by feeling able to reframe their own experiences and counter any negative beliefs and attitudes associated with the diagnosis that they may have internalised over time (Chan and Tsui 2025).

Finally, as a person with a personality disorder themselves, PSWs also stand to benefit from working within mental health services. Being able to support individuals experiencing similar challenges to their own, and using their experience and knowledge to have a positive impact on another service user, can go on to make PSWs feel more empowered in their own recovery (Wilson 2024). They will be more likely to engage in self‐compassion and self‐care, supporting their own recovery as well as the recovery of others.

### 4.4 Strengths and Limitations

A range of databases were used, as well as checking the reference lists of the included studies, which allowed us to identify a mix of qualitative and quantitative studies to inform our discussions. Two reviewers (RBM and SG) carried out full‐text screening, data extraction and quality assessment, and any conflicts were then resolved through discussion, reducing the risk of bias of the findings.

However, several of the included studies had discussed limitations associated with their sample, such as small/limited sample sizes, which can affect the generalisability of the data (Jewell et al. 2022; Cawood 2012; Turner et al. 2024; Barr et al. 2022; D'Sa and Rigby 2011; Seal et al. 2024). Furthermore, some of the included studies had majority or all female samples, and so it cannot be reliably determined if these findings would be relevant to men with personality disorder. Despite this, it could be argued that this skewed sample may be representative of the clinical reality of personality disorder, with women with personality disorder being more likely to present to services (Dyer 2016; Paris 2004).

### 4.5 Clinical and Research Implications

The findings of this review highlight the need to evaluate service delivery for people with personality disorder. At present, the role of PSWs appears to be ambiguous and requires greater clarity to enable their successful integration into MDTs, ultimately moving away from a service model in which clinical perspectives are viewed as superior to one where lived experience is also valued and appreciated. For this to be successful, services must also allocate sufficient resources to the training and development of PSWs, as well as reducing professional stigma towards personality disorder. Challenging professional stigma and subsequent negative attitudes towards PSWs who have this lived experience is vital in providing compassionate mental healthcare. By seeking to confront such attitudes, mental health services stand to benefit from peer support interventions and the benefits they can provide to patients. Developing and empowering PSWs has the potential to transform the care being received by service users, supporting them in their recovery journey and increasing the likelihood of successful treatment outcomes.

The results of this review also highlight the need for further research in this area. Fully powered clinical trials are needed to evaluate the costs and benefits of the deployment of PSWs in services for people with personality disorder. Such studies should follow up participants beyond the treatment period and include outcome measures that are endorsed by people with personality disorder (Prevolnik Rupel et al. 2021). Trials with active control groups would ensure that the extent of added value that results from support from PSWs would be fully elicited.

## 5 Conclusion

This systematic review examined the barriers, facilitators and impact of using PSWs in services for people with personality disorder. PSWs can take on varied roles in the support they provide, including facilitating support groups, providing validation and empathy, modelling recovery and advocating for service users. However, for successful integration, services need to provide clear role definitions, robust training and supervision and challenge stigmatising attitudes among professionals.

While the evidence base is still developing, these findings suggest that peer support could be a valuable addition to specialist personality disorder services if meaningfully implemented. Further research with larger, more diverse samples is needed to strengthen the evidence, and services should begin to consider how they may be able to effectively integrate PSWs into services with the aim of enhancing care for people with personality disorder.

## Funding

The authors have nothing to report.

## Conflicts of Interest

The authors declare no conflicts of interest.

## Supporting information


**Appendix S1:** Search strategy.

**Appendix S2:** Quality assessment.

## Data Availability

The data that support the findings of this study are available from the corresponding author upon reasonable request.
