# Supplementary material for: Implementation and Outcomes of Peer Support Workers in Services for People With Personality Disorder: A Systematic Review and Narrative Synthesis
Source: Personal Ment Health. 2026 Mar 8;20(2):e70066. doi: 10.1002/pmh.70066 (PMC12967695; doi:10.1002/pmh.70066)
Supplement: Supplementary file 1 — Appendix S1: Search strategy. [file PMH-20-0-s002.docx]

***Appendix 1- Search Strategy***

| **Database** | **Date last searched** | **Full search strategy** | **Total records identified** |
| --- | --- | --- | --- |
| Medline (Ovid) | 15/08/2025 | (("peer$" or "expert by experience$" or "expert patient$") and ("personality disorder$" or "complex emotional needs$")).mp. [mp=title, book title, abstract, original title, name of substance word, subject heading word, floating sub-heading word, keyword heading word, organism supplementary concept word, protocol supplementary concept word, rare disease supplementary concept word, unique identifier, synonyms, population supplementary concept word, anatomy supplementary concept word] | 969 |
| Embase (Ovid) | 15/08/2025 | (("peer$" or "expert by experience$" or "expert patient$") and ("personality disorder$" or "complex emotional needs$")).mp. [mp=title, abstract, heading word, drug trade name, original title, device manufacturer, drug manufacturer, device trade name, keyword heading word, floating subheading word, candidate term word] | 1,009 |
| PsycINFO (Ovid) | 15/08/2025 | (("peer$" or "expert by experience$" or "expert patient$") and ("personality disorder$" or "complex emotional needs$")).mp. [mp=title, abstract, heading word, table of contents, key concepts, original title, tests & measures, mesh word] | 1,131 |
| PubMed | 15/08/2025 | ("peer$" OR "expert by experience$" OR "expert patient$") AND ("personality disorder$" OR "complex emotional needs$")  ("peer"[All Fields] OR "expert by experience"[All Fields] OR "expert patient"[All Fields]) AND ("personality disorder"[All Fields] OR "complex emotional needs"[All Fields]) | 661 |
| CINAHL | 15/08/2025 | (“peer$” OR “expert by experience$” OR “expert patient$”) AND (“personality disorder$” OR “complex emotional needs$”)  Expanders - Apply equivalent subjects  Search modes - Proximity | 199 |
