# Supplementary material for: Implementation and Outcomes of Peer Support Workers in Services for People With Personality Disorder: A Systematic Review and Narrative Synthesis
Source: Personal Ment Health. 2026 Mar 8;20(2):e70066. doi: 10.1002/pmh.70066 (PMC12967695; doi:10.1002/pmh.70066)
Supplement: Supplementary file 2 — Appendix S2: Quality assessment. [file PMH-20-0-s001.docx]

***Appendix 2- Quality assessment***

RANDOMISED CONTROLLED TRIALS

|  | **Grenyer et al. (2025)** | **Mahlke et al. (2017)** |
| --- | --- | --- |
| Was true randomization used for assignment of participants to treatment groups? | Yes | Yes |
| Was allocation to treatment groups concealed? | No | Yes |
| Were treatment groups similar at the baseline? | Yes | Yes |
| Were participants blind to treatment assignment? | No | No |
| Were those delivering the treatment blind to treatment assignment? | No | No |
| Were treatment groups treated identically other than the intervention of interest? | Yes | Yes |
| Were outcome assessors blind to treatment assignment? | No | No |
| Were outcomes measured in the same way for treatment groups? | Yes | Yes |
| Were outcomes measured in a reliable way? | Yes | Yes |
| Was follow up complete and if not, were differences between groups in terms of their follow up adequately described and analysed? | Yes | No |
| Were participants analysed in the groups to which they were randomized? | Yes | Yes |
| Was appropriate statistical analysis used? | Yes | Yes |
| Was the trial design appropriate and any deviations from the standard RCT design (individual randomization, parallel groups) accounted for in the conduct and analysis of the trial? | Yes | Yes |

**Table 1:** Quality assessment for randomised controlled trials, The revised JBI critical appraisal tool for the assessment of risk of bias for randomised controlled trials (Barker et al., 2023)

UNCONTROLLED PRE-POST STUDIES

|  | **Blay et al. (2025)** | **Jewell et al. (2022)** |
| --- | --- | --- |
| Is it clear in the study what is the “cause” and what is the “effect”? | Yes | Yes |
| Was there a control group? | No | No |
| Were participants included in any comparisons similar? | N/A | N/A |
| Were the participants included in any comparisons receiving similar treatment/care, other than the exposure or intervention of interest? | N/A | N/A |
| Were there multiple measurements of the outcome, both pre and post the intervention/ exposure? | Yes | Yes |
| Were the outcomes of participants included in any comparisons measured in the same way? | Yes | Yes |
| Were outcomes measured in a reliable way? | Yes | Yes |
| Was follow-up completed and if not, were differences between groups in terms of their follow-up adequately described and analysed? | Yes |  |
| Was appropriate statistical analysis used? | Yes | Yes |

**Table 2:** Quality assessment for uncontrolled pre-post studies, The revised JBI critical appraisal tool for the assessment of risk of bias for quasi-experimental studies (Barker et al., 2024)

CROSS-SECTIONAL STUDIES

|  | **Cawood (2012)** | **Turner et al. (2024)** |
| --- | --- | --- |
| Were the criteria for inclusion in the sample clearly defined? | Yes | Yes |
| Were the study subjects and the setting described in detail? | Yes | Yes |
| Was the exposure measured in a valid and reliable way? | Yes | Yes |
| Were objective, standard criteria used for measurement of the condition? | Yes | No |
| Were confounding factors identified? | No | No |
| Were strategies to deal with confounding factors stated? | No | No |
| Were the outcomes measured in a valid and reliable way? | Yes | Yes |
| Was appropriate statistical analysis used? | Yes | Yes |

**Table 3:** Quality assessment for cross-sectional studies (Moola et al., 2020)

QUALITATIVE STUDIES

|  | **Barr et al. (2022)** | **Barr et al. (2020)** | **Crawford et al. (2007)** | **Dahlenburg et al. (2024)** | **D’Sa & Rigby (2011)** | **Gillard et al. (2015)** | **Seal et al. (2024)** |
| --- | --- | --- | --- | --- | --- | --- | --- |
| Is there congruity between the stated philosophical perspective and the research methodology? | Yes | Yes | N/A | N/A | N/A | Yes | N/A |
| Is there congruity between the research methodology and the research question or objectives? | Yes | Yes | Yes | Yes | Yes | Yes | Yes |
| Is there congruity between the research methodology and the methods used to collect data? | Yes | Yes | Yes | Yes | Yes | Yes | Yes |
| Is there congruity between the research methodology and the representation and analysis of data? | Yes | Yes | Yes | Yes | Yes | Yes | Yes |
| Is there congruity between the research methodology and the interpretation of results? | Yes | Yes | Yes | Yes | Yes | Yes | Yes |
| Is there a statement locating the researcher culturally or theoretically? | No | Yes | Yes | No | No | Yes | No |
| Is the influence of the researcher on the research, and vice-versa, addressed? | No | No | Yes | No | No | Yes | No |
| Are participants, and their voices, adequately represented? | Yes | Yes | Yes | Yes | Yes | Yes | Yes |
| Is the research ethical according to correct criteria or, for research studies, and is there evidence of ethical approval by an appropriate body? | Yes | Yes | Yes | Yes | Yes | Yes | Yes |
| Do the conclusions drawn in the research report flow from the analysis, or interpretation, of the data? | Yes | Yes | Yes | Yes | Yes | Yes | Yes |

**Table 4:** Quality assessment for qualitative studies (Lockwood et al., 2015)
